# Procedures Relative to the Single Care of Insane Persons

**Published:** 1900-08-11

**Authors:** F. St. John Bullen


### Hospital Clinics and Medical Progress.

### "PROCEDURES RELATIVE TO THE SINGLE CARE OF INSANE PERSONS."

By F. St. John Bullen, M.R.C.S.

(*Continued from page* 284.)

The discharge of a patient has usually been discussed and agreed upon by the person in charge and the practitioner, but the latter may at any time order the discharge of the patient, and, unless the medical attendant certify in writing that the patient is unfit to be at large, this order must be complied with. The medical attendant may, on his part, warn the petitioner of the patient's recovery, and such notice demands the removal of the same within seven days. A physical examination should be made and noted previous to removal, and a notice of the discharge should be promptly sent to the Commissioners. In the case of death a notice and certain particulars must be sent to the coroner by the medical attendant not later than 48 hours after decease. A notice of death has also to be sent to the district registrar, Commissioners, principal relative named in the certificates, and to the petitioner. A copy of the medical statement as regards the death must be inserted in the journal. Restraint may, under circumstances of urgency, be needed, and may be straightway applied by the person in charge, but notice of this must be given as soon as possible to the medical attendant, whose duty it will be to enter full particulars of the surgical or medical reasons, excessive destructiveness, &c., of the patient, which alone justify the use of this measure. A copy of the entries, comprising times of application, duration, and method employed must be sent to the Commissioners every quarter by the person in charge, and all apparatus used for restraint be ready for production to the Commissioners on their visit. Any frequency in the application of restraint would probably cause the Commissioners to order the removal of the patient to an institution. It may happen that a medical man is summoned to visit a person who is suffering from acute symptoms of insanity, and who is placed in such circumstances that there is no friend or relative responsible for his care. If the case be urgent it may be dealt with in the following ways. The medical man may himself sign the statement for an urgency order and obtain a medical certificate from another doctor with whom he has no working or other connection; or he may make the certificate himself and procure a statement from the proprietor of the dwelling in which the patient is, or a minister of religion, &c. This procedure will authorise admission to a licensed house, or a pauper asylum where private cases are received, or charge in a medical man's house, and also legalises measures for restraint. Another method is to treat the patient as a person "not under proper care and control," and to call in a relieving officer, overseer, or constable, by whose authority the patient may be temporarily placed in the workhouse until removal to an asylum can be arranged. This last course is the one probably best to take, inasmuch as a private or public institution will not generally receive a private patient without a written guarantee of payment for a quarter, which the medical man or person acting may reasonably decline to subscribe to. All, then, that either of these need do is to call in the parish official and indicate that the patient is "a lunatic not under proper care and control." It is, however, not always possible to find in a short time the relieving officer or his substitute, and these, again, or the constable may not regard the conditions under which the patient is placed as justifying action on this plea. By agreement with the official, however, the case may be dealt with under the heading of a "lunatic wandering at large," by releasing the patient in a public thoroughfare and immediately apprehending him. Where no institution to which he can be taken is near, then detention for the night in the police-station can be arranged. Whenever possible, friends or relatives of the patient should be searched for and communicated with before removal to an institution, since they may choose to assume charge. Upon giving a satisfactory undertaking to care for and properly guard the patient, the justice who has signed the order for the patient's detention in an institution can accept their responsibility, and so also can any two of the visitors to the asylum in which it has been arranged to place the patient. If he has been admitted to an asylum the visitors must be applied to for a discharge or transfer order, supposing this to be desirable.

Other points regarding certificates are these. The subject of amendments has been alluded to. The various forms contain marginal notes which give all particulars if carefully read, but a few additional hints may be offered. The seven days between the date of interviewing the patient and the presentation to the justice of the petition are clear days, and are counted exclusively of both the dates of the interview and of the presentation. The petitioner must have seen the patient within 14 days before the presentation of the petition, and here the day of the interview should be reckoned in and that of presentation excluded, so that the petition may be presented on the fifteenth day after making it—Sundays, Christmas Day, Good Friday, and public holidays must be included.

The various forms required in dealing with the charge of a single patient, and affecting every contingency, are obtainable from Shaw and Son, Fetter Lane, E.C. A manuscript book with the few requisite headings written by hand is sufficient for the Medical Journal, and so also with the Case Book and Restraint Register.

The foregoing sketch is intended to indicate the duties of a medical man who wishes to assume the care of an insane person, and he would do well in mastering the contents of Dr. Mercier's handbook of Lunacy Law should he undertake the charge.

